# Butanediammonium Salt Additives for Increasing Functional and Operando Stability of Light-Harvesting Materials in Perovskite Solar Cells

**DOI:** 10.3390/nano12244357

**Published:** 2022-12-07

**Authors:** Natalia N. Udalova, Aleksandra K. Moskalenko, Nikolai A. Belich, Pavel A. Ivlev, Andrey S. Tutantsev, Eugene A. Goodilin, Alexey B. Tarasov

**Affiliations:** 1 Laboratory of New Materials for Solar Energetics, Department of Materials Science, Lomonosov Moscow State University, Lenin Hills, 119991 Moscow, Russia; 2 Department of Chemistry, Lomonosov Moscow State University, Lenin Hills, 119991 Moscow, Russia

**Keywords:** perovskite solar cells, hybrid halide perovskites, exploitation stability, defect passivation, diammonium cations, layered lead halide perovskites, bulk passivation, BDAI_2_, 1,4-butanediammonium iodide

## Abstract

Organic diammonium cations are a promising component of both layered (2D) and conventional (3D) hybrid halide perovskites in terms of increasing the stability of perovskite solar cells (PSCs). We investigated the crystallization ability of phase-pure 2D perovskites based on 1,4-butanediammonium iodide (BDAI_2_) with the layer thicknesses n = 1, 2, 3 and, for the first time, revealed the presence of a persistent barrier to obtain BDA-based layered compounds with n > 1. Secondly, we introduced BDAI_2_ salt into 3D lead–iodide perovskites with different cation compositions and discovered a threshold-like nonmonotonic dependence of the perovskite microstructure, optoelectronic properties, and device performance on the amount of diammonium additive. The value of the threshold amount of BDAI_2_ was found to be ≤1%, below which bulk passivation plays the positive effect on charge carrier lifetimes, fraction of radiative recombination, and PSCs power conversion efficiencies (PCE). In contrast, the presence of any amount of diammonium salt leads to the sufficient enhancement of the photothermal stability of perovskite materials and devices, compared to the reference samples. The performance of all the passivated devices remained within the range of 50 to 80% of the initial PCE after 400 h of continuous 1 sun irradiation with a stabilized temperature of 65 °C, while the performance of the control devices deteriorated after 170 h of the experiment.

## 1. Introduction

The steep progress of perovskite photovoltaics over the last decade relies mainly on the impressive growth of record power conversion efficiencies up to 25.7%, being almost as high as the record PCE of monocrystalline silicon-based solar cells (26.1%) [1]. At the same time, the long-term stability of PSCs is still far from the target value of 20–25 years [2], despite significant progress in this field driven by a number of approaches like perovskite compositional engineering [3], dopant-free hole-transporting materials [4], barrier layers [5], the application of 2D perovskites [6], and 2D/3D heterostructures [7], as well as the control of surface and bulk defects in perovskite materials and interfaces through passivation with various organic or inorganic compounds [8]. Organic ammonium salts have been successfully used in the last three approaches [9,10,11]. The most prominent member of these additives is the n-butylammonium (BA^+^) cation, which forms a homologous row of layered (BA)_2_(MA)_n−1_Pb_n_X_3n+1_ perovskites with a wide range of *n* [12]. The use of n-butylammonium-based 2D/3D heterostructures allows to create devices with record open-circuit voltage [13] and outstanding exploitation stability [14]. Along with such clear advantages, monoammonium cations demonstrate a tendency of dynamical evolution of 2D/3D interfaces under heating and light illumination, leading to PSC instability and loss of performance [15,16].

Diammonium cations should be considered as a promising alternative with various advantages, including their much lower deprotonation ability, the absence of van der Waals gaps in 2D perovskite structures and possible influence on perovskite grain and domain interconnections [6,17]. Indeed, a direct stability comparison of PSCs based on monoammonium and diammonium cations revealed the superiority of diammonium counterparts used as either spacer cations in 2D perovskites [6,18] or passivating agents in 3D structures [19]. It is known that different chemical structures and carbon chain lengths of diammonium cations play a crucial role in PSCs performance and stability [20,21,22], thus demanding a proper selection of organic compounds with desired functional groups. One of the record PSC stabilities over 5000 h under continuous 1 sun illumination has been achieved so far by using 2D perovskites with a 1,3-propanediammonium (PDA) spacer cation and cesium co-doping [23]. On the other hand, a direct comparison of diammonium cations with different chain lengths within 2D perovskites, provided by Zheng et al., revealed the superior performance and stability of PSCs with a 1,4-butanediammonium (BDA) spacer cation, as compared to PDA-based devices [21]. The same trend was reported for the case of surface passivation with diammonium halides, where BDAI_2_ demonstrated better performance than PDAI_2_ [22]. These results are reliable, since PDAI_2_ (in contrast to BDAI_2_) forms a non-perovskite structure with the empirical formula [PDAPbI_4_]_15_•[PDAI_2_] [24]. Therefore, 1,4-butanediammonium appears to be a more promising alternative to shorter PDA^2+^ cations and other diammonium counterparts. However, there are very few works regarding the use of BDA^2+^ in PSCs and the available results are controversial in terms of the ability to crystallize different members of the 2D (BDA)(MA)_n−1_Pb_n_I_3n+1_ compositional row; BDA^2+^ localization in 3D perovskites; the role of BDA^2+^ additives in performance and photothermal stability of perovskite solar cells.

Here, we provided a systematic study on the use of 1,4-butanediammonium diiodide (BDAI_2_) either as a spacer cation in 2D (BDA)(MA)_n−1_Pb_n_I_3n+1_ perovskites or as a passivating agent in 3D MA_0_._25_FA_0_._75_PbI_3_ and FA_0_._85_Cs_0_._15_PbI_3_ counterparts to define the most effective method of introducing BDAI_2_ into light-harvesting materials in perovskite solar cells.

## 2. Materials and Methods

**Materials.** Methylammonium iodide (CH_3_NH_3_I = MAI; 99%, Dyesol, Queanbeyan, Australia), formamidinium iodide (CH(NH_2_)_2_I = FAI; ≥99%, Dyesol), cesium iodide (CsI; >99%, TCI, Tokyo, Japan), butane-1,4-diammonium iodide (INH_3_(CH_2_)_4_NH_3_I = BDAI_2_, ≥99%, Dyesol), lead iodide (PbI_2_; >98%, Lanhit, Moscow, Russia), n-butylammonium iodide (C_4_H_9_NH_3_I = BAI; ≥99%, Dyesol), chlorobenzene (C_6_H_5_Cl = CB; anhydrous, 99.8%, Sigma-Aldrich, St. Louis, MO, USA), dimethylsulfoxide ((CH_3_)_2_SO = DMSO; anhydrous, ≥99.9%, Sigma-Aldrich), N,N-dimethylformamide (HCON(CH_3_)_2_ = DMF; anhydrous, 99.8%, Sigma-Aldrich), toluene (anhydrous, 99.8%, Sigma-Aldrich), propylene carbonate (≥99.9%, Sigma-Aldrich), methylammonium chloride (CH_3_NH_3_Cl = MACl; ≥99%, Merck, Rahway, NJ, USA), fluorine-doped tin oxide (FTO) substrates (FTO-coated glass slide, ∼7 Ω/sq, Sigma-Aldrich), Spiro-OMeTAD (99.5%, Sigma-Aldrich), 4-tert-butylpyridine (TBP, >96%, Sigma-Aldrich), LiTFSI (99.8%, Sigma-Aldrich), anhydrous acetonitrile (99.8% Alfa Aesar, Haverhill, MA, USA), FK209 (tris(2-(1H-pyrazol-1-yl)-4-tert-butylpyridine)cobalt(III) tris[bis(trifluoromethane)sulfonimide], 98%, Sigma-Aldrich), Au (99.95%), molecular iodine, molybdenum trioxide (MoO_3_, 99.99%, Lanhit), titanium (IV) isopropoxide (97%, Sigma Aldrich), acetilacetone (99.5%, Sigma Aldrich), isopropyl alcohol (99.8%, Component-Reactiv), mercaptoacetic acid (98%, Acros) and SnCl_2_·2H_2_O (99.99%, Lanhit) were commercially purchased.

**Synthesis of 2D perovskite crystals.** Crystals with the following nominal compositions were synthesized by the solution method described in [25]: BA_2_PbI_4_, BA_2_MAPb_2_I_7_, (BDA)PbI_4_, and (BDA)MAPb_2_I_7_. A 1.5M stoichiometric solution of the corresponding halide salts in a mixture of propylene carbonate and hydroiodic acid was kept at a constant temperature of 70 °C for 8 h. A (BDA)MAPb_2_I_7_ solution was also prepared with a 1.5 M and 0.1 M concentration to study the effect of concentration on 2D perovskite phase composition. Crystallization in the (BDA)MAPb_2_I_7_ solution with 25, 50, 75, and 100% excess of MAI, with respect to Pb content, was achieved with 1.5 M solutions using the same method. All the resulting crystals were dried, ground, and studied by powder X-ray diffraction.

**Crystallization of (BDA)MAPb_2_I_7_ and (BDA)MA_2_Pb_3_I_10_ using an equilibrium system with a transfer mediator.** To study the equilibrium phases in the (BDA)(MA)_n−1_Pb_n_I_3n+1_ system with n = 2 and 3, we carried out the following experiment: the mixtures of solid-state BDAI_2_ + MAI + PbI_2_ precursors with (BDA)(MA)Pb_2_I_7_ (n = 2) and (BDA)(MA)_2_Pb_3_I_10_ (n = 3) overall compositions were thoroughly ground in an argon-filled glove box (<10 ppm O_2_; 0.1 ppm H_2_O) and then dispersed in dry toluene with a 5% amount of molecular iodine as a mass-transfer agent. The 5% amount of iodine was estimated with respect to the total lead amount. The prepared solid–liquid mixtures were continuously stirred and heated at 70 °C for 7 days to achieve thermodynamic equilibrium. After the resulting powders were washed several times with pure toluene, they were dried, and again ground in the glove box with dry air (<20% RH) just before XRD analysis.

**Deposition of 3D perovskite films.** MA_0_._25_FA_0_._75_PbI_3_ perovskite films were prepared from 1.5 M solutions of MAI, FAI, PbI_2_ in stoichiometric molar ratios with 15% excess of MACl in DMF/DMSO (4:1 *v*/*v*) via 1-step antisolvent spin-coating in an inert glove box. Spin-coating was performed at 6000 rpm for 30 s, where 100 µL of chlorobenzene was added dropwise at the 5th second of rotation. The following annealing step was performed at 125 °C for 20 min: FA_0_._85_Cs_0_._15_PbI_3_ perovskite films were prepared by the same method from 1.3 M stoichiometric solution of FAI, CsI, and PbI_2_ in DMF/DMSO (4:1 *v*/*v*). The spin-coating method was the same as in the previous example, except for the addition of CB at the 15th second of rotation. Annealing was also performed for 20 min at 125 °C.

Bulk passivation of both perovskite compositions was carried out following the addition of 0.2, 0.5, 1, 2.5 and 5 mol.% BDAI_2,_ with respect to Pb content, to the initial perovskite solutions by mixing two perovskite solutions with 0 and 5% BDAI_2_ in the volume ratios that corresponded to the resulting amount of the additive.

**Fabrication of perovskite solar cells.** FTO-coated glass substrates were patterned via chemical etching (Zn powder + 4 M HCl) and then sequentially cleaned with detergent (2% Hellmanex solution), distilled water, and the prepared solution of concentrated H_2_SO_4_ + 30% H_2_O_2_ in a volume ratio of 3:1 for 10 min. After this, the substrates were washed in deionized water, dried by N_2_ gas flow, and immediately transferred onto a hotplate for the deposition of the blocking TiO_2_ layer. A compact TiO_2_ layer was formed by spray pyrolysis at 450 °C. The precursor solution was prepared by mixing 900 μL of titanium (IV) isopropoxide, 600 μL of acetilacetone and 50 mL of isopropyl alcohol. After spraying, the substrates were annealed for 60 min at 450 °C and then left to cool down. The deposition of the SnO_2_ layer was carried out according to the method stated in ref. [26]. Shortly after, 0.625 g of urea was dissolved in 500 mL of deionized water, followed by the addition of 10 μL of mercaptoacetic acid and 0.5 mL of 37% HCl. Finally, 0.1 g of SnCl_2_·2H_2_O was dissolved in the above-mentioned solution. The glass/FTO/c-TiO_2_ substrates were previously treated by ozone with UV/ozone cleaner (Osilla) for 15 min, placed vertically into a glass with the as-prepared solution, and then stored in a laboratory oven at 70 °C for 3 h. After SnO_2_ deposition, the substrates were rinsed with deionized water and sonicated for 2 min to remove large agglomerates from the SnO_2_ film surface. Finally, the dried substrates were transferred onto a hotplate and annealed at 180 °C for 1 h. FA_0_._85_Cs_0_._15_PbI_3_ + *x*% BDAI_2_ perovskite films (where *x* = 0.25, 0.5, 1, 2.5, and 5%) were deposited on FTO/c-TiO_2_/SnO_2_ substrates, according to the previously described protocol. The spiro-OMeTAD solution was prepared by dissolving 60 mg of Spiro-OMeTAD in 598 μL of CB with 21.6 μL of TBP, 12 μL of 1.8 M solution of LiTFSI in anhydrous acetonitrile, and 4.8 μL of 0.25 M solution of co-complex FK209 in anhydrous acetonitrile. Deposition of 28 mkl of doped spiro-OMeTAD solution was carried out at 4000 rpm in an inert glove box. Finally, an Au electrode with ~80 nm thickness was deposited by thermal evaporation (base pressure 10^−5^ Torr) through a shadow mask.

**Encapsulation of perovskite solar cells.** The method for the encapsulation of PSCs was provided by ref. [27]. At the first stage, a 150–200 nm layer of molybdenum trioxide (MoO_x_) was thermally evaporated under a high vacuum (*P* = 1 × 10^−5^ mbar) on top of the as-prepared PSCs. The deposition rate was monitored via QSM sensors and maintained at 0.4–0.7 Å/s. After the evaporation of the capping layer, the solar cells were immediately transferred into a nitrogen-filled glovebox. The second encapsulation step was carried out with commercially available UV-curable polymers (“Nano Clear” by FunToDo) and a cover glass slide. For this step, 5–7 μL of epoxy was dispensed onto an active area of each device and covered by a glass slide. Immediately after this, the photopolymerization of the epoxy was carried out via UV light exposure (10 W InGaAlN LED; λ = 365–375 nm) for 1–2 min.

**Perovskite solar cell characterization and stability measurements.** The current density–voltage (J–V) characteristics of solar cells were measured with a Keithley series 2450 source meter under a simulated sun AM 1.5 G (ORIEL LSH-7320 ABA LED Solar Simulator) with custom-built python-based software. The light intensity was calibrated to 1 sun (AM1.5, 100 mWcm^−2^) by unfiltered Si reference cells calibrated by Newport.

The long-term photostability of encapsulated PSCs under ambient conditions was achieved using an unfiltered sulfur plasma lamp (LG PSH 0731B), calibrated with Si reference cells to a 100 ± 10 mW/cm^2^ power density. To maintain the desired device temperature, PSCs were placed onto a custom-built copper stage that was consistently heated to 65 °C. MPPT was performed using a standard perturb and observe algorithm and the voltage was updated every 5 min with a 10 mV step by a homemade electronic board with 16-channel MPPT capability.

**X-Ray powder diffraction.** XRD analysis was performed using a Bruker Advance D8 diffractometer with Bragg–Brentano geometry and Cu Kα radiation (λ = 15,418 Å). XRD patterns were recorded in the 3–35° 2θ range with 0.1 s per dot and a 0.02° step.

**SEM.** Scanning electron microscopy (SEM) was performed on a Zeiss Supra 40 microscope with an accelerating voltage of 1–5 kV.

**PL spectroscopy.** PL spectra were recorded via a home-built setup based on Thorlabs components with a 405 nm excitation laser, 10x focusing objective lens, and an Ocean Optics Flame spectrometer. The laser power was ∼200–210 μW, and the laser spot size was ∼10 μm.

**Time-resolved photoluminescence spectroscopy.** Time-resolved photoluminescence spectroscopy (TRPL) was performed with a laser pulse frequency of 1000 kHz and a single pulse time of 30 ps. PL decay curves were recorded on a calibrated HMP-100-50 single-photon detector (Becker and Hickl, Germany), equipped with an SPC-150 module. The PL decay curves were processed using the SPC Image software (Becker and Hickl) and OriginPro. To approximate the decay curves, we used the sum of two exponential decay functions, which was as follows: 𝜏_avg_ = (A_1_𝜏_1_ + A_2_𝜏_2_)/(A_1_ + A_2_), where A_i_ and 𝜏_I_ are the amplitude and lifetime of charge carriers for the ith component of the decay curve, respectively.

**Photostability study of perovskite films.** The photostability study of perovskite films was carried out according to the protocol described in [28]. The perovskite films on FTO substrates were irradiated with a white LED with 100 mW/cm^2^ power density in an argon-filled glove box (<10 ppm O_2_). All perovskite films were irradiated from the substrate side to minimize the interference of defective perovskite surfaces in light-induced degradation [29]. The characterization of perovskite films was periodically provided by PL spectroscopy under an inert atmosphere. The comparison of the photostability of different perovskite samples was carried out based on normalized PL intensity values as a function of the light-soaking time.

## 3. Results and Discussion

The synthesis of higher n members of (BDA)(MA)_n−1_Pb_n_I_3n+1_ 2D perovskites has not been yet clearly presented, while published XRD and optical spectroscopy data are very contradictory [18,30]. Often, authors discuss the problem of the solution synthesis of phase-pure (BDA)(MA)_n−1_Pb_n_I_3n+1_ with n > 1, demonstrating the formation of multiple-phase samples with the prevailing (BDA)PbI_4_ and/or 3D perovskite phases [31,32,33]. An analysis of the open access 2D perovskites database [34] reveals that only (BDA)PbI_4_ (n = 1) crystallographic data have been reported [35].

**Figure 1:**
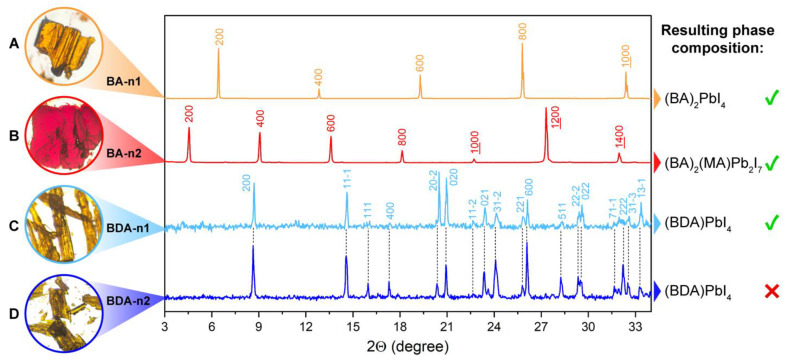
Optical photographs of single crystals, synthesized from solutions relevant to (BA)_2_PbI_4_ (**A**), (BA)_2_(MA)Pb_2_I_7_ (**B**), (BDA)PbI_4_ (**C**), (BDA)(MA)Pb_2_I_7_; (**D**) perovskite compositions and corresponding powder XRD patterns. On the right, the actual phase composition of each sample is given, indicating the failure to obtain the (BDA)(MA)Pb_2_I_7_ compound.

To clarify this issue, we tried to synthesize (BDA)(MA)_n−1_Pb_n_I_3n+1_ layered perovskites with n = 2 and 3 using the following two methods: crystallization from a 1.5 M solution of nPbI_2_ + (n-1)MAI + BDAI_2_ in an HI + propylene carbonate mixture, as previously reported in [25], and slow crystallization using an equilibrium system with a transfer mediator. At first, we achieved the solution synthesis of single crystals for (BDA)(MA)_n−1_Pb_n_I_3n+1_ and (BA)_2_(MA)_n−1_Pb_n_I_3n+1_ (BA—butylammonium) compositions with n = 1 and 2 (Figure 1). In the case of BA-based solutions, high-quality single crystals with target n numbers were successfully obtained (Figure 1A,B), while only the (BDA)PbI_4_ phase (yellow crystals) was able to crystallize from BDA-based solutions, regardless of the initial precursors’ stoichiometry (Figure 1C,D). This behavior of the system was previously explained by the much lower solubility of the n = 1 member, due to the strong electrostatic affinity of diammonium cations towards inorganic perovskite-like layers [31]. Additional attempts to crystallize (BDA)(MA)Pb_2_I_7_ perovskites from a diluted 0.1 M solution also resulted in the formation of (BDA)PbI_4_ phases (Figure S1). Hypothetically, the excess of MAI in the solution that corresponds to the (BDA)(MA)Pb_2_I_7_ composition could shift the equilibrium of the system towards the crystallization of the n = 2 member. However, according to our experimental results, the BDA-based solution system does not respond to the variation in MAI:BDAI_2_ stoichiometry up to 2:1 (100% excess of MAI), thus preserving (BDA)PbI_4_ phase composition (Figure S2).

**Figure 2:**
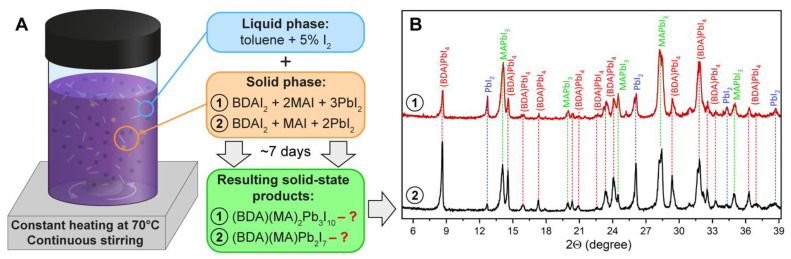
(**A**) The scheme of the experimental setup for the study of reactivity of the mixture of BDAI_2_ + MAI + PbI_2_ powders with component ratios corresponding to (BDA)(MA)_2_Pb_3_I_10_ (1) and (BDA)(MA)Pb_2_I_7_ (2). The role of component transfer media in 5% I_2_ solution in dry toluene. (**B**) XRD patterns of the resulting solid-state products after 7 days of continuous heating and stirring of the initial precursor mixtures. Each peak is labeled by the corresponding phase: (BDA)PbI_4_ (red), PbI_2_ (blue); MAPbI_3_ (green).

To study the thermodynamic equilibrium in the (BDA)(MA)_n−1_Pb_n_I_3n+1_ system, we carried out the following experiment: the mixtures of solid-state BDAI_2_ + MAI + PbI_2_ precursors with (BDA)(MA)_2_Pb_3_I_10_ (n = 3) and (BDA)(MA)Pb_2_I_7_ (n = 2) overall compositions were thoroughly ground in an inert glove box and then dispersed in dry toluene with a 5% amount of molecular iodine as a mass-transfer agent (Figure 2A). The mass transfer proceeds through the liquid media via the formation of liquid alkylammonium polyiodides [36,37], which play the role of highly reactive intermediate phases that quickly yield hybrid iodoplumbates with the highest thermodynamic stability. The grinding stage can initiate the solid-state formation of layered perovskite phases, which was reported earlier as a more perspective preparation method for diammonium-based 2D perovskites than solution synthesis [31]. The prepared solid–liquid mixtures were continuously stirred and heated at 70 °C for 7 days; after this, the resulting powders were washed several times with pure toluene, dried, and again ground just before XRD analysis. Diffraction patterns of the resulting powder samples are given in Figure 2B, demonstrating that the mixtures of (BDA)PbI_4_ and MAPbI_3_ with some amount of unreacted PbI_2_ formed instead of (BDA)(MA)_2_Pb_3_I_10_ and (BDA)(MA)Pb_2_I_7_ phases in both cases. A relative amount of (BDA)PbI_4_ and MAPbI_3_ phases correlates with the initial stoichiometry; the (BDA)PbI_4_ phase dominates the (BDA)(MA)Pb_2_I_7_ sample (Figure 2B(2)), while 3D perovskite is the main product for the (BDA)(MA)_2_Pb_3_I_10_ sample (Figure 2B(1)).

Considering these results along with previously reported data, we conclude that the formation of a light-absorbing layer based on phase-pure 2D (BDA)(MA)_n−1_Pb_n_I_3n+1_ perovskite is unfavorable. On the other hand, passivation of 3D perovskites with BDAI_2_ appears to be more beneficial and can be accompanied by the formation of either (BDA)PbI_4_ phases or BDA^2+^ layers at the interfaces and/or perovskite intra and intergrain boundaries (GBs). In the case of surface passivation, the formation of (BDA)PbI_4_ phases on the perovskite surface was previously reported [38,39,40], but the effect of bulk passivation with BDAI_2_ on the structure and optical properties of 3D perovskites has not been fully discussed, despite the potential for more efficient passivation of cation and anion vacancies throughout the thickness of the perovskite film. To clarify this issue, we performed bulk passivation of MA_0_._25_FA_0_._75_PbI_3_ (MAFAPI) and FA_0_._85_Cs_0_._15_PbI_3_ (FACsPI) perovskite films with different amounts of BDAI_2_ and studied the effect of passivation on the morphology, crystal structure, optical properties, and photostability of perovskite films, as well as PSCs. The use of mixed-cation perovskites was motivated by their higher PCE and stability compared to the conventional MAPbI_3_ composition [41,42,43,44]. The amount of diammonium additives in the perovskite solution varied in a wide range from trace 0.025 wt.% [17] to 3–8% [20,45]; therefore, we chose the following BDAI_2_ concentrations for 3D perovskite bulk passivation: 0.25, 0.5, 1, 2.5, and 5%.

**Figure 3:**
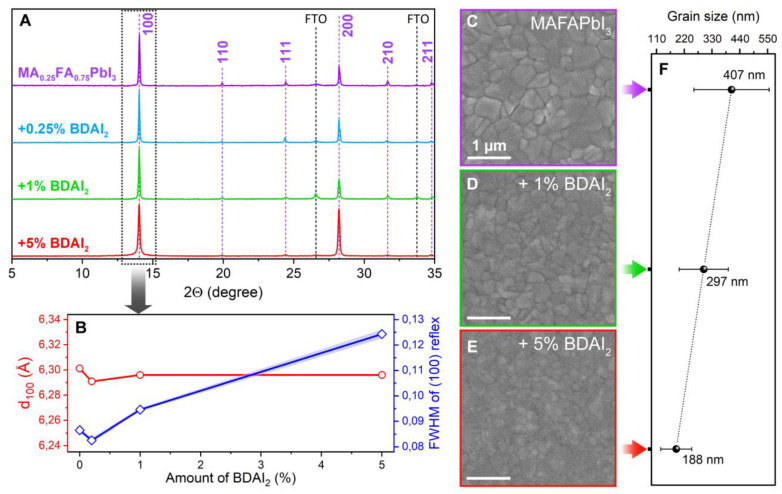
(**A**) XRD patterns of MA_0_._25_FA_0_._75_PbI_3_ perovskite films with a different amount of BDAI_2_ additive. (**B**) Interplanar distance (red) and FWHM (blue) of (100) perovskite reflex as a function of BDAI_2_ amount. (**C**–**E**) Top-view SEM images of MAFAPI perovskite films without additive (**C**), with 1% BDAI_2_ (**D**), and with 5% BDAI_2_ (**E**). The scale bars are 1 µm. (**F**) Average grain size of corresponding perovskite films with different BDAI_2_ contents.

According to the XRD data of the MA_0_._25_FA_0_._75_PbI_3_ + *x*% BDAI_2_ samples (Figure 3A), no phase impurities, such as (BDA)PbI_4_ or delta polytypes of FAPbI_3_, formed after the introduction of BDAI_2_ into the material. The same behavior was observed for the FACsPI + *x*% BDAI_2_ samples (Figure S3). Interplanar distances in the perovskite lattice were also not affected by the diammonium additive (Figure 3B). Evidently, such small amounts of additives would not be detected by XRD; however, we observed the widening of diffraction reflexes along with an increased amount of the BDAI_2_ additive, as shown in Figure 3B for the (100) perovskite reflex. This seems to correlate with the monotonic decrease in the average grain size from 407 ± 149 nm for the reference MAFAPI sample, down to 188 ± 46 nm for MAFAPI + 5% BDAI_2_ (Figure 3C–F). In the case of FACsPI perovskite composition, we also observed a pronounced decrease in grain size with the addition of ≥1% BDAI_2_, while 0.25% of diammonium salt does not noticeably affect the perovskite morphology (Figure S4). The decrease in grain size with the BDAI_2_ additive may be due to the preferential localization of diammonium cations on the GBs instead of the perovskite lattice, since a too large BDA^2+^ cation cannot be incorporated into 3D perovskites without severe lattice distortions. Similar trends with decreasing grain size during perovskite passivation with diammonium salts are reported elsewhere [45,46].

**Figure 4:**
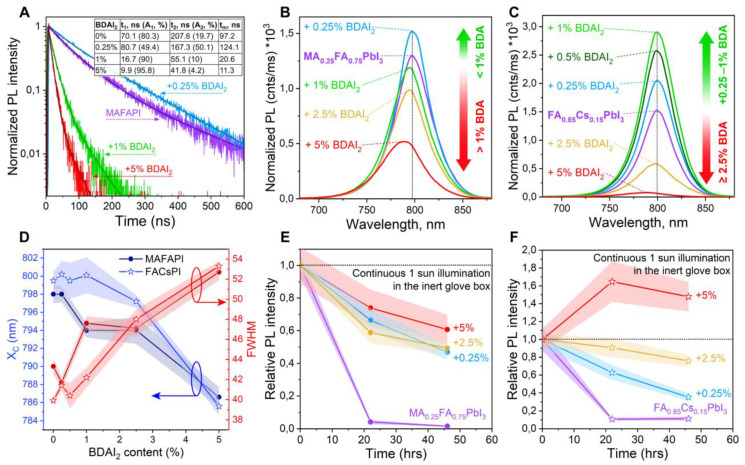
(**A**) Time-resolved PL decay curves of MA_0_._25_FA_0_._75_PbI_3_ + *x%* BDAI_2_ films. Estimated charge carrier lifetimes (t_1_ and t_2_ with corresponding relative amplitudes A_1_ and A_2_, and average lifetime t_m_) are given in the inset table in (**A**). (**B**,**C**) Steady-state PL spectra of MA_0_._25_FA_0_._75_PbI_3_ + *x%* BDAI_2_ (**B**) and FA_0_._85_Cs_0_._15_PbI_3_ + *x%* BDAI_2_ (**C**) films. (**D**) Graphical illustration of PL peak positions (X_C_) and widths (FWHM) as a function of BDAI_2_ content for both MAFAPI (–●–) and FACsPI (–✩–) samples. (**E**,**F**) Photostability of MAFAPI (**E**) and FACsPI (**F**) films with different BDAI_2_ contents as an evolution of PL intensity, under continuous 1 sun irradiation in inert glove box.

The optoelectronic properties of perovskite films also depend strongly on the BDAI_2_ amount. Time-resolved photoluminescence (TRPL) data of the MAFAPI + *x*% BDAI_2_ samples reveal a dramatic decrease in the average charge carrier lifetimes t_m_ from 97.2 ns for the reference perovskite sample down to 20.6 ns and 11.3 ns for the samples with 1% and 5% BDAI_2_, respectively (Figure 4A). The lifetime decrease is accompanied by the growing proportion of fast decay components (t_1_ in the inset table in Figure 4A), which are usually associated with trap-assisted recombination on the surface and at the GBs. In contrast, a small amount of diammonium additive (0.25% BDAI_2_) leads to an increase in the average lifetime t_m_ up to 124,1 ns and a simultaneous rise in the slow decay component t_2_, which is thought to originate from perovskite bulk [47]. Steady-state photoluminescence (PL) data of MAFAPI + *x*% BDAI_2_ correlate with the TRPL decay curves; perovskite PL intensity increases with 0.25% BDAI_2_ with respect to the reference MAFAPI sample, but is progressively quenched with the further addition of 1, 2.5, and 5% BDAI_2_ (Figure 4B). Interestingly, FACsPI perovskite composition has higher “tolerance” to the BDAI_2_ additive, with a gradual increase in PL intensity up to 1% BDAI_2_ (Figure 4C). After overcoming the threshold level of 1% BDAI_2_, FACsPI films also suffer from a dramatic loss of PL intensity, which is accompanied by the widening of emission lines (Figure 4D, red lines). In addition, the center of the PL peaks (X_C_) of both perovskite compositions shifts to shorter wavelengths up to 14 nm at the maximum BDAI_2_ content of 5% (Figure 4D, blue lines). Similar trends are observed for the FACsPI + x% BDAI_2_ samples by optical absorption spectroscopy (Figure S5). This slight increase in the perovskite bandgap could be explained by the distortions of the inorganic [PbI_3_]^–^ sublattice [46], which is predominantly located at grain boundaries [48], the proportion of which becomes higher with the increasing amount of diammonium additive according to the morphological observations (Figure 3C–F and Figure S4).

The comparative study of long-term photostability of MAFAPI and FACsPI perovskite films was carried out in accordance with the previously reported approach based on continuous light soaking of perovskite films in an inert glove box and periodic registration of PL signals [28]. The resulting evolution of relative PL intensity of the perovskite samples, normalized to the first measurements, is given in Figure 4E for MAFAPI and Figure 4F for FACsPI compounds. The reference samples of both perovskites undergo a dramatic drop in PL intensity after 22 h of light soaking, as 0.25% of the BDAI_2_ additive is sufficient to slow down the degradation process. The further increase in the BDAI_2_ content up to 5% reveals a different response of MAFAPI and FACsPI perovskites’ photostability to bulk passivation. The MAFAPI films show no further stabilization of the PL signal, demonstrating threshold-like behavior and the effectiveness of small amounts of diammonium salt for the improvement of both perovskite optoelectronic properties and long-term photostability. In turn, the FACsPI perovskite composition appears to be more sensitive to BDAI_2_ content with a photostability that is proportional to the passivator amount (Figure 4F). FACsPI + 5% BDAI_2_ demonstrates enhancement of the PL signal under continuous 1 sun illumination, while 2.5% of the additive preserves 80% of the initial PL intensity. These observations, together with the steady-state PL data, motivate us to choose the FACsPI perovskite composition with BDAI_2_ bulk passivation as a light absorbing material in PSCs.

**Figure 5:**
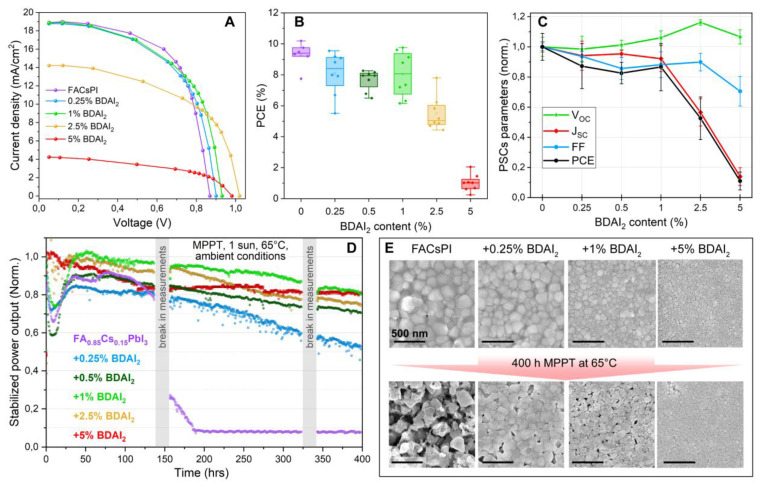
(**A**) Quasi-steady-state J–V curves of record FA_0_._85_Cs_0_._15_PbI_3_-based devices with 0.25, 1, 2.5, and 5% BDAI_2_ additive. (**B**) Distribution of absolute PCE values of FA_0_._85_Cs_0_._15_PbI_3_-based PSCs with a different BDAI_2_ content. (**C**) The dependence of FF, V_OC_, J_SC_, and PCE parameters as a function of BDAI_2_ content. Each parameter is normalized by the corresponding average value of the reference devices. (**D**) Normalized values of stabilized power output (SPO) of encapsulated devices with a different BDAI_2_ amount. Vertical grey regions illustrate the technical interruptions in signal registration. During this time, light soaking and heating did not stop; the devices were loaded at a voltage near the MPP conditions. (**E**) SEM images of FACsPI + x% BDAI_2_ perovskite surface in PSCs before (top row) and after 400 h of MPPT at 65 °C (bottom row). All scale bars correspond to 500 nm.

Hence, we investigated the influence of BDAI_2_ passivation on FACsPI-based PSC performance and stability. The devices were assembled with the following planar architecture: FTO/c-TiO_2_/SnO_2_/perovskite/spiro-OMeTAD/Au and encapsulated in an inert glove box with MoO_x_ and epoxy resin in accordance with ref. [27]. The analysis of J–V curves (Figure 5A), PCE statistics (Figure 5B) and dynamics of other PSC working parameters (Figure 5C) as a function of BDAI_2_ content reveals again the threshold-like behavior of the devices, where >1% BDAI_2_ content initiates an almost order of magnitude PCE decrease for the 5% BDAI_2_ additive, while ≤1% BDAI_2_ has a slight effect on PCE (Figure 5A,B). A comparative analysis of basic PSC working parameters, including fill factor (FF), open circuit voltage (V_OC_), short circuit current density (J_SC_), and PCE (Figure 5C), normalized to the corresponding values of the control devices, reveals a strong correlation of PCE with J_SC,_ which agrees with the previously observed decrease of the average grain sizes and charge carrier lifetimes in perovskite films (Figure 3C–F and Figure S4). The absolute values of all the listed parameters are given in the Supporting Information (Figure S6). The fill factor of PSCs tends to monotonically decrease, losing up to 30% of the initial FF value, in contrast to the increasing V_OC_. Usually, a V_OC_ increase is explained by the reduction in the trap densities in the device [49], and also correlates with a slight widening of the perovskite bandgap with the addition of ≥1% BDAI_2_ (Figure S5 and Figure 4D).

We also estimated the series and shunt resistances from quasi-steady-state J–V curves and revealed similar trends, as both resistances tended to increase with growing amounts of diammonium additive (Figure S7). It means that BDAI_2_ passivation exerts two opposite effects on hybrid perovskite materials and devices, including the positive effect of decreasing the number of shunts and the negative effect of decreasing the electric conductivity in the devices.

The long-term stability tests of encapsulated PSCs were performed under constant ∼100 mW/cm^2^ white light illumination and a stabilized temperature of 65 °C, using a maximum power point tracking (MPPT) regime in an ambient environment. The resulting stabilized power outputs (SPO) of the best devices with different BDAI_2_ contents, normalized to the first measurements, are shown in Figure 5D. The reference FACsPI-based solar cell undergoes rapid irreversible degradation between 140 and 190 h of the experiment, being the least stable device in the row. This is accompanied by the severe deterioration of the initial perovskite morphology (Figure 5E). In contrast, all passivated solar cells demonstrate superior stability, remaining within the 50 to 80% range of the initial SPO after 400 h of continuous photothermal aging. The least stable device within this group appears to be FACsPI + 0.25% BDAI_2_, while all other samples with more diammonium content demonstrate higher stability. The absolute values of SPO are provided in SI (Figure S8). Importantly, the presence of BDAI_2_ even at a low level of 0.25% protects the perovskite morphology and phase from degradation on a timescale of 400 h, which is illustrated by the comparative SEM images in Figure 5E. The enlarged version of these microscopy data is given in Figure S9. Interestingly, we observed unusual behavior of the SPO during the first 50 h of the light-soaking experiment with a pronounced drop in the device performance, which was restored to the initial value (in the case of 1% BDAI_2_) or to 80–90% of the initial SPO (for reference, 0.25%, and 0.5% BDAI_2_ samples) (Figure 5D). In the literature, similar SPO evolution was previously reported for n-i-p PSCs architectures by Jung et al. [50] and Bai et al. [51] without, however, any explanation. Notably, high enough BDAI_2_ contents (≥2.5%) diminish this effect.

We suppose that the mechanism of PSC stability increase with BDAI_2_ passivation could originate from the intergrain “encapsulation” by either (BDA)PbI_4_ phases or BDA^2+^ monolayers and the additional improvement of the interconnection between perovskite grains, owing to bidentate diammonium cations. This hypothesis is primarily supported by the electron microscopy data (Figure 5E), as well as by the increasing values of shunt resistance in the passivated devices (Figure S7). The presence of diammonium passivation at the GBs, which are known to be the main channels of ion migration [52], could also decrease the extent of extrinsic and intrinsic ionic migration—the main factor of potential-induced degradation in various types of solar cells, including PSCs [53].

## 4. Conclusions

In conclusion, we experimentally demonstrated that (BDA)PbI_4_ is the only stable 2D perovskite phase based on 1,4-butanediammonium cations, which means that (BDA)(MA)_n−1_Pb_n_I_3n+1_ compositional row is not suitable for the use as phase-pure light-harvesting materials in PSCs. The bulk passivation of 3D ABX_3_ hybrid perovskites with BDAI_2_ leads to the modulation of the perovskites’ microstructure and optoelectronic properties depending on the amount of diammonium additive. BDA^2+^ cations seem to accumulate mostly on the perovskite grain boundaries in the form of either (BDA)PbI_4_ phases or BDA^2+^-containing layers, leading to the gradual decrease in the average grain size with an increased BDAI_2_ content. No phase impurities were detected in the passivated samples. The influence of BDAI_2_ passivation on perovskite optoelectronic properties and device performance demonstrates a threshold behavior with a positive effect on perovskite materials below the threshold (increase in PL intensity, charge carrier lifetimes, V_OC_; shunt resistance) and a strongly negative effect above the threshold (decrease in PL intensity and charge carrier lifetimes, J_SC_ decrease; series resistance increase). In the case of BDAI_2_, the threshold value was found to be ~ 1 mol.% for FACsPI and ~ 0.5 mol.% for MAFAPI compositions, with respect to Pb content. On the other hand, the presence of any amount of diammonium salt leads to the sufficient enhancement of the photothermal stability of perovskite materials and devices, compared to the reference samples. The performance of the passivated devices remained within the 50 to 80% range of the initial PCE after 400 h of continuous 1 sun irradiation with a stabilized temperature of 65 °C, while the performance of the control devices deteriorated after 170 h of the experiment. The most possible stabilization mechanism of BDAI_2_-passivated PSCs is assumed to originate from the intergrain encapsulation by either (BDA)PbI_4_ phases or BDA^2+^ monolayers and the additional improvement of interconnection between perovskite grains, owing to bidentate diammonium cations. Therefore, 1,4-butanediammonium iodide could be considered as a promising additive for the improvement of PSC stability, but the amount of this salt should be carefully controlled to improve the performance of the final devices.

## Data Availability

Not applicable.

## Supplementary Materials

The following supporting information can be downloaded at https://www.mdpi.com/article/10.3390/nano12244357/s1: Figure S1: XRD patterns of 2D perovskites, prepared from (BDA)(MA)Pb_2_I_7_ solutions with different concentrations; Figure S2: XRD patterns of 2D perovskite crystals, prepared from (BDA)(MA)Pb_2_I_7_ solutions with different excess amounts of MAI; Figure S3: XRD patterns of FA_0_._85_Cs_0_._15_PbI_3_ perovskite films with different BDAI_2_ contents; Figure S4: SEM images of FACsPI perovskite films with different BDAI**_2_** contents; Figure S5: Tauc plots of FA_0_._85_Cs_0_._15_PbI_3_ perovskite films with different BDAI_2_ contents; Figure S6: Distributions of operando parameters of PSCs based on FACsPI + *x*% BDAI_2_ light-harvesting materials; Figure S7: The dependence of R**_series_** and R**_shunt_** of PSCs on BDAI_2_ content; Figure S8: Absolute SPO values of encapsulated perovskite solar cells with different BDAI_2_ amounts under continuous 1 sun illumination, stabilized temperature of 65 °C, and ambient environment; Figure S9: SEM images of FACsPI perovskite films with different BDAI_2_ contents before and after 400 h of PSC photothermal aging.
