# Supplementary material for: Butanediammonium Salt Additives for Increasing Functional and Operando Stability of Light-Harvesting Materials in Perovskite Solar Cells
Source: Nanomaterials (Basel). 2022 Dec 7;12(24):4357. doi: 10.3390/nano12244357 (PMC9784390; doi:10.3390/nano12244357)
Supplement: Supplementary file 1 [file nanomaterials-12-04357-s001.zip › nanomaterials-2061149-supplementary.pdf]

Butanediammonium Salt Additives for Increasing Functional and Operando Stability of Light-Harvesting Materials in Perovskite Solar Cells

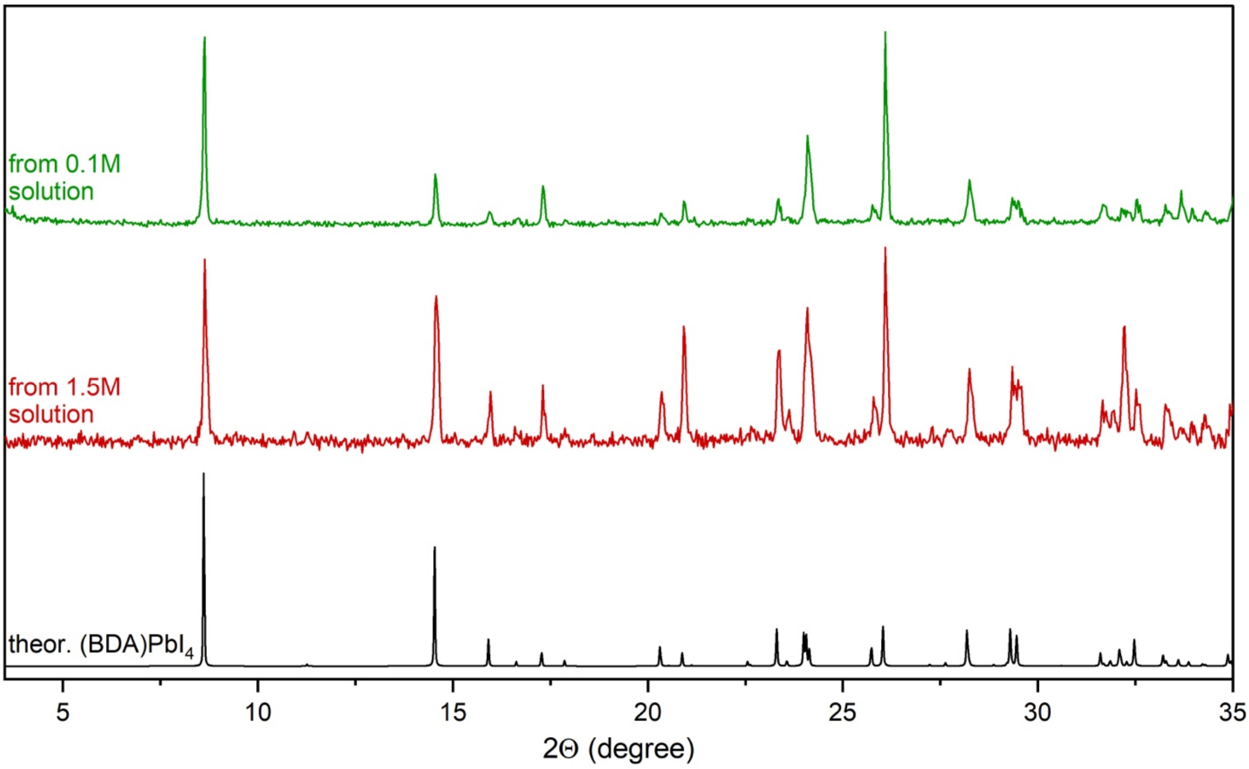

Figure S1. Powder XRD patterns of 2D perovskite crystals, prepared from 0.1M (green line) and 1.5M (red line) (BDA)(MA)Pb_2I_7 solutions. Black line corresponds to theoretical XRD pattern of (BDA)PbI_4 phase.

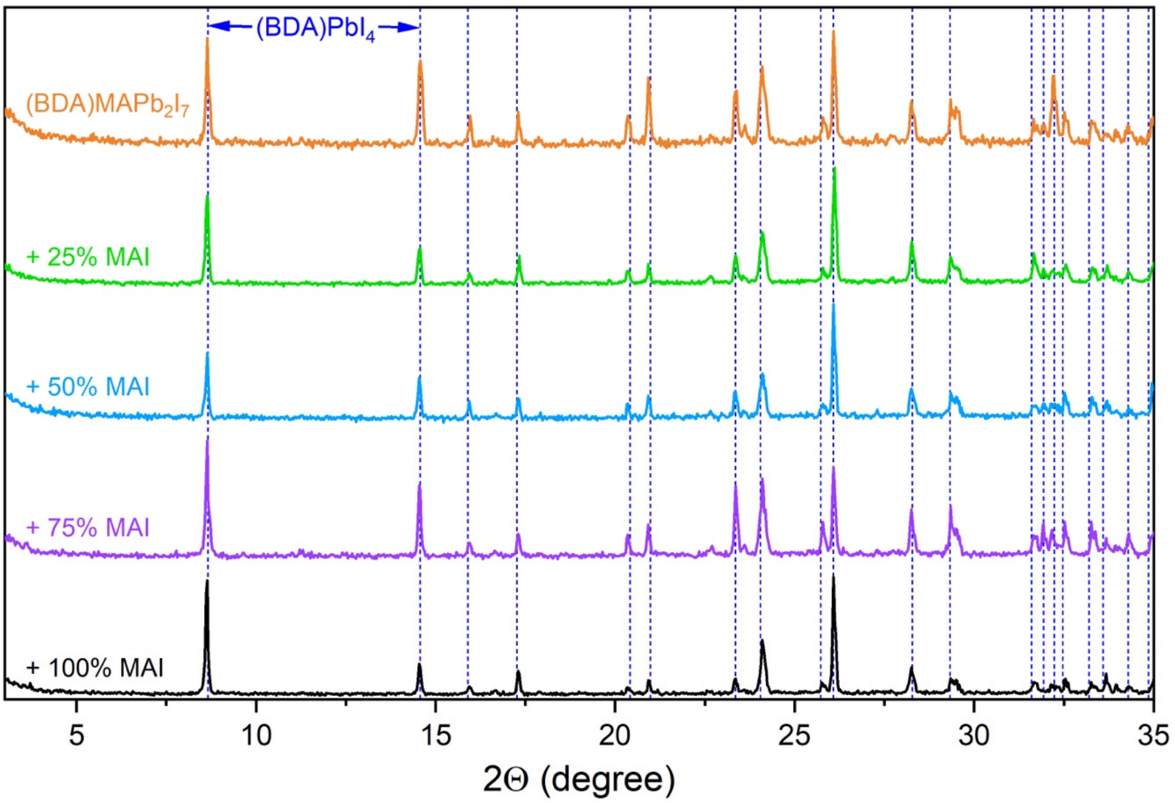

Figure S2. Powder XRD patterns of 2D perovskite crystals, prepared from stoichiometric (BDA)(MA)Pb_2I_7 solution (upper line) and from solutions with different excess of MAI, specified above each line. Vertical dashed lined correspond to the reflexes of (BDA)PbI_4 phase.

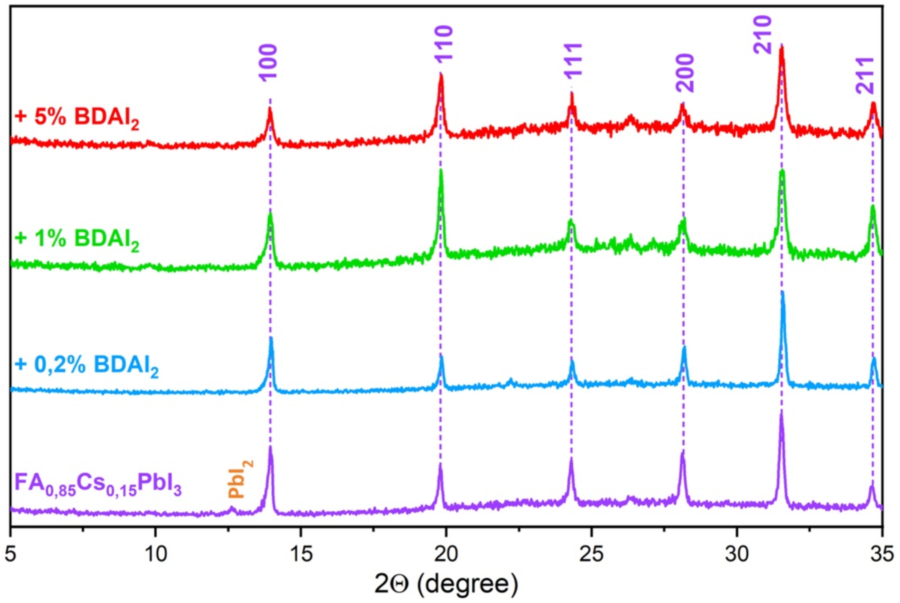

Figure S3. XRD patterns of $FA_{0.85}Cs_{0.15}PbI_3$ perovskite films with different $BDAI_2$ content.

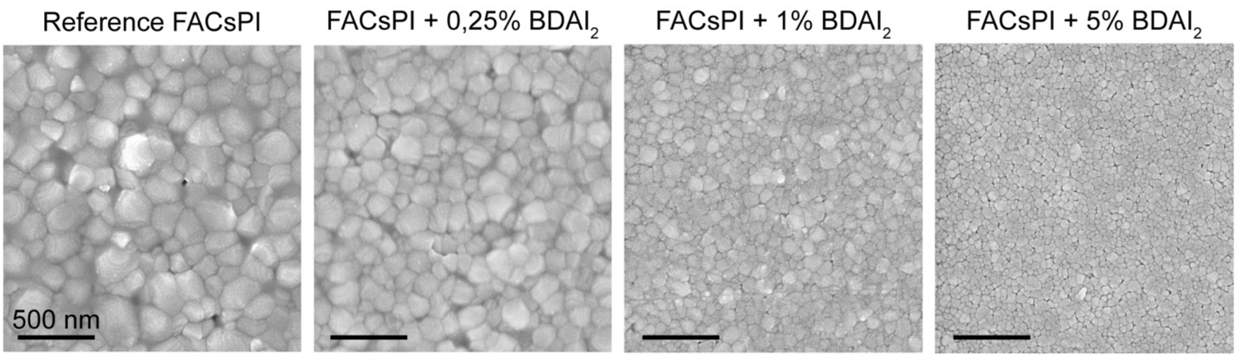

Figure S4. SEM images of FACsPI perovskite films with 0, 0.25, 1, and 5% $BDAI_2$ content.

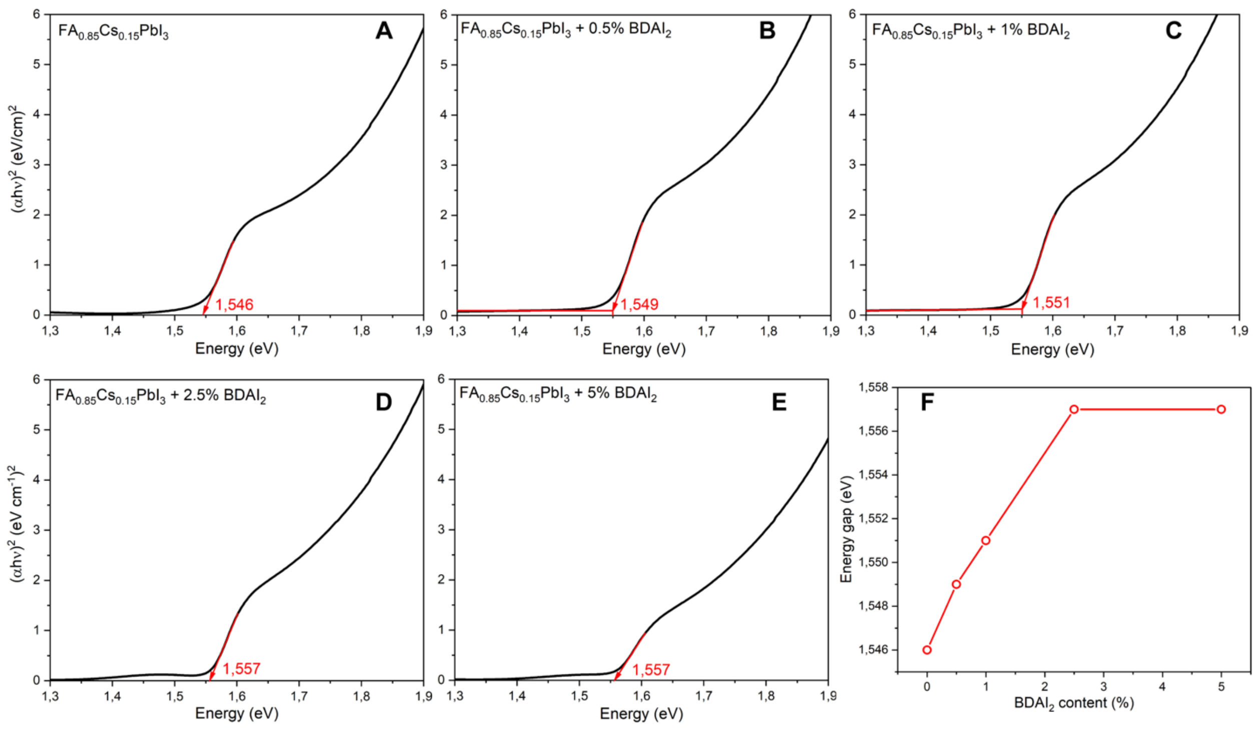

Figure S5. (A-E) Tauc plots of $FA_{0.85}Cs_{0.15}PbI_3$ perovskite films with different $BDAI_2$ content. (F) Energy gap of $FA_{0.85}Cs_{0.15}PbI_3$ perovskite as a function of $BDAI_2$ content, calculated from corresponding Tauc plots.

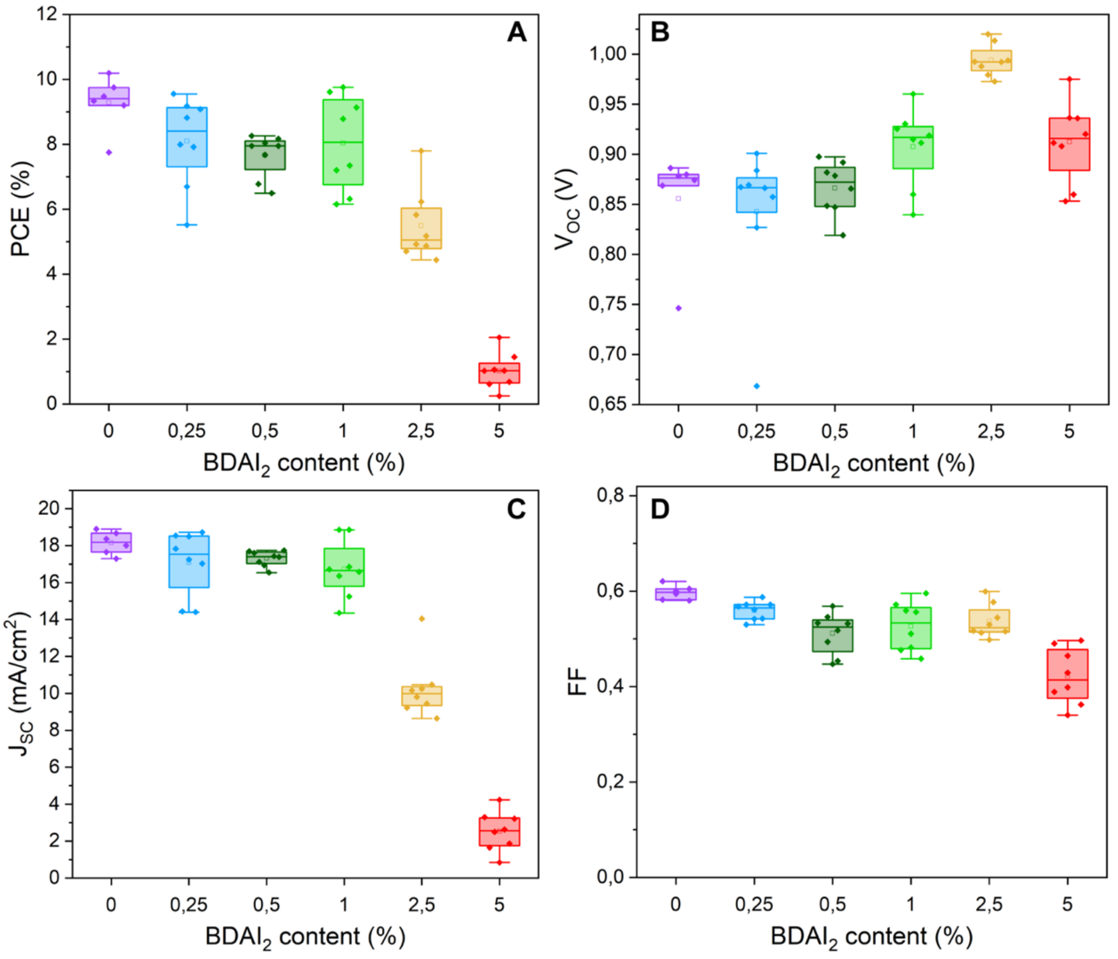

Figure S6. Distributions of operando parameters of PSCs based on FACsPI + *x*% $BDAI_2$ light-harvesting materials: PCE (A), V_{OC} (B), J_{SC} (C), and FF (D).

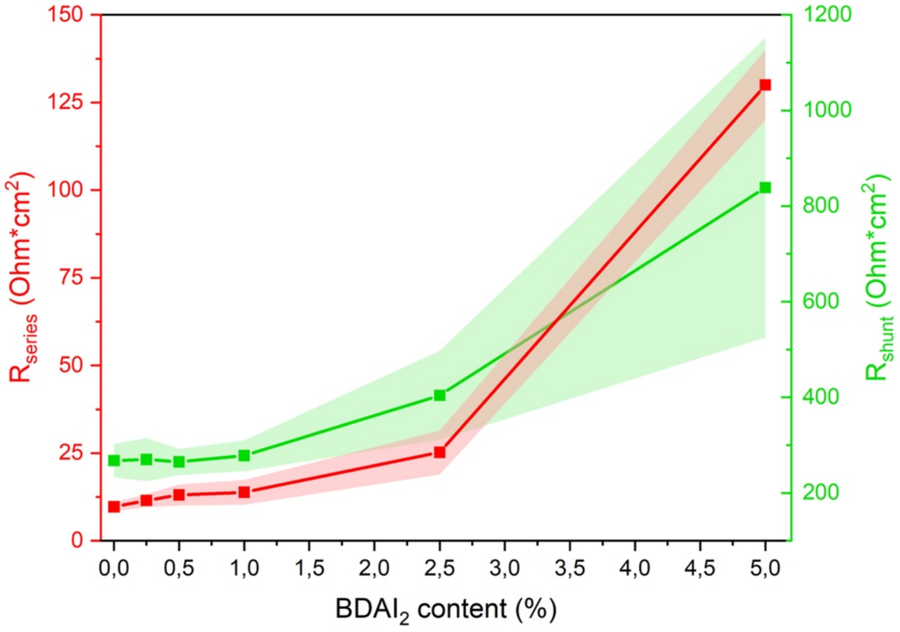

Figure S7. The dependence of series resistance (red) and shunt resistance (green) of PSCs on $BDAI_2$ content, estimated from quasi steady state J-V curves under 1 sun illumination.

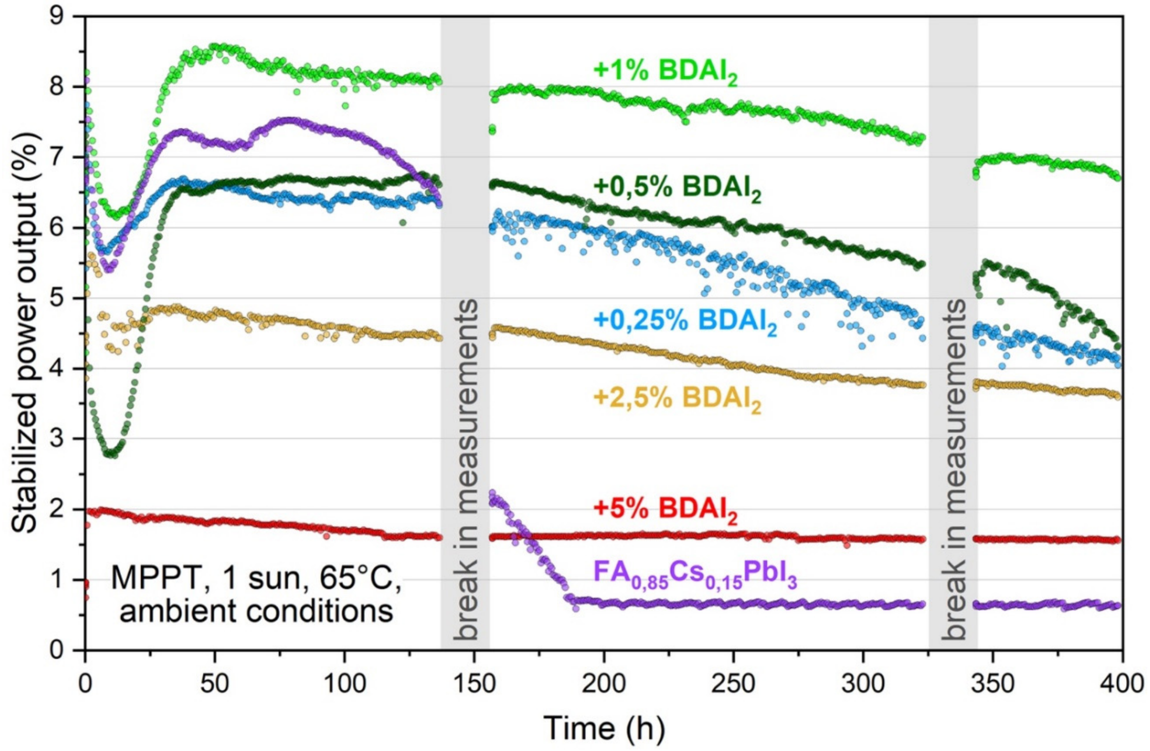

Figure S8. Stabilized power output of encapsulated perovskite solar cells with different $BDAI_2$ amounts under continuous 1 sun illumination, stabilized temperature 65°C, and ambient environment. Twice during the experiment SPO measurements were technically interrupted for several hours with preserved light and heat aging, PSCs during these periods were loaded close to MPP.

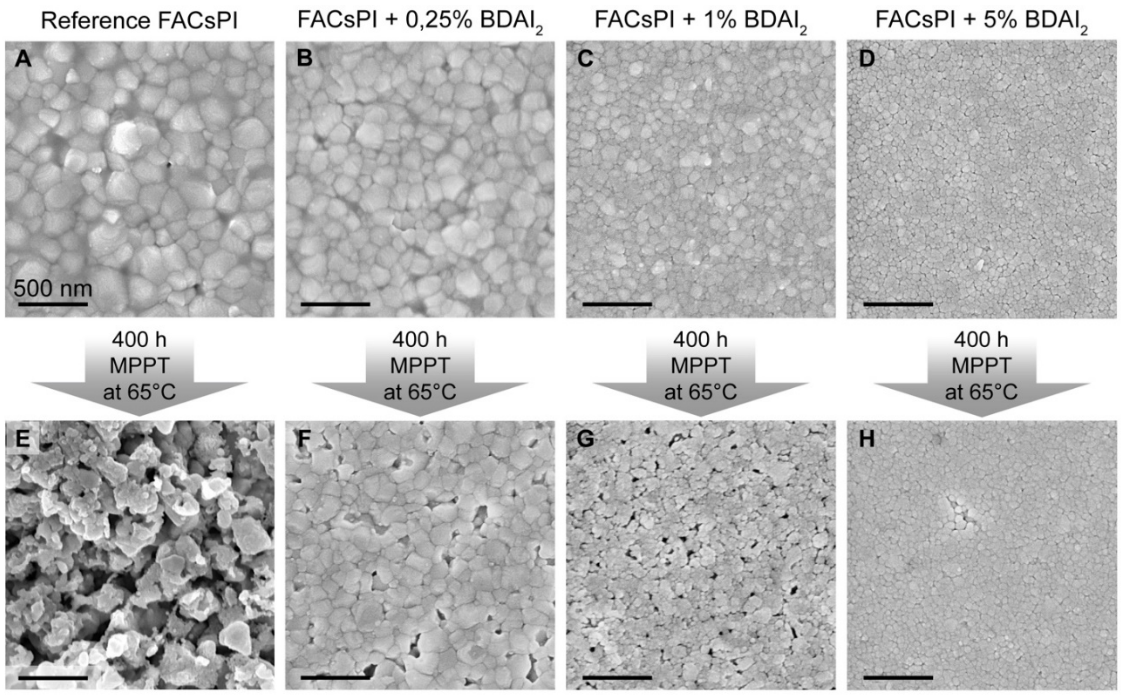

Figure S9. SEM images of four FACsPI perovskite films with different $BDAI_2$ content: 0% (A, E), 0.25% (B, F), 1% (C, G), and 5% (D, H) before (A-D) and after (E-H) 400 hours of MPPT under constant 1 sun illumination and 65°C. All scale bars are 500 nm.
